# SARS-CoV-2 Genetic Variability and Non-Specific Immunity Associated with the Use of Different BCG Strains—A Molecular and Clinical Approach

**DOI:** 10.3390/vaccines9060639

**Published:** 2021-06-10

**Authors:** Jakub Kulus, Magdalena Kulus, Katarzyna Stefańska, Jarosław Sobolewski, Hanna Piotrowska-Kempisty, Paul Mozdziak, Bartosz Kempisty

**Affiliations:** 1 Department of Diagnostics and Clinical Sciences, Institute of Veterinary Medicine, Nicolaus Copernicus University in Torun, 87-100 Torun, Poland; jakub.kulus@umk.pl; 2 Department of Veterinary Surgery, Institute of Veterinary Medicine, Nicolaus Copernicus University in Torun, 87-100 Torun, Poland; magdalena.kulus@umk.pl; 3 Department of Histology and Embryology, Poznan University of Medical Sciences, 60-781 Poznan, Poland; k.stefanska94@o2.pl; 4 Department of Public Health Protection and Animal Welfare, Institute of Veterinary Medicine, Nicolaus Copernicus University in Torun, 87-100 Torun, Poland; jsobolewski@umk.pl; 5 Department of Toxicology, Poznan University of Medical Sciences, 60-631 Poznan, Poland; hpiotrow@ump.edu.pl; 6 Department of Basic and Preclinical Sciences, Institute of Veterinary Medicine, Nicolaus Copernicus University in Torun, 87-100 Torun, Poland; 7 Prestage Department of Poultry Science, College of Agriculture and Life Sciences, North Carolina State University, Raleigh, NC 27695, USA; pemozdzi@ncsu.edu; 8 Department of Anatomy, Poznan University of Medical Sciences, 60-781 Poznan, Poland

**Keywords:** SARS-CoV-2, COVID-19, BCG vaccinations, genetic variability, WHO recommendation, variants, tuberculosis

## Abstract

The effect of BCG vaccination against tuberculosis on the reduction in COVID-19 infection is related to the effect of the BCG vaccine on the immunomodulation of non-specific immunity. In the early stages of the pandemic, countries with universal BCG vaccination programs registered a low number of new cases of COVID-19, with the situation now reversed, as exemplified by India. The high genetic variability of SARS-CoV-2, a known characteristic of RNA viruses, causing the occurrence of SARS-CoV-2 variants may have led to the virus adapting to overcome the initial immune protection. The strains from the United Kingdom (B1.1.7), Brazil (B1.1.28 and B1.1.33), South Africa (B.1.351), and India (B.1.617) are characterized by a greater ability to spread in the environment, in comparison with the original infectious agent of SARS-CoV-2. It should be remembered that the large variation in the genetic makeup of SARS-CoV-2 may result in future changes in its pathogenicity, immunogenicity and antigenicity, and therefore it is necessary to carefully study the mutations occurring within the virus to determine whether the current vaccines will remain effective. However, most studies show that monoclonal antibodies produced after vaccination against COVID-19 are effective against the newly developed variants.

## 1. Introduction

The current coronavirus disease (COVID-19) pandemic has affected the whole world since 2020. Despite prevalent and commercially available COVID-19 vaccination programs being in place, several scientists are still conducting research into the already available drugs and vaccines for this infectious disease.

The Bacillus Calmette–Guérin (BCG) vaccine remains an interest of a number of scientists due to its beneficial nonspecific effects on the immune system [1]. The study by Escobar et al. [2], consistent with other epidemiological studies [3,4], suggests an association between a lower number of cases and diminished mortality from COVID-19 with the presence of national mandatory BCG vaccination programs. However, one of the most substantial limitations of this type of study is a lack of credible data and the presence of numerous confounding variables.

Health system underfunding in low- and middle-income countries [5] may result in reduced COVID-19 testing capacity and a low level of confirmed cases [6]. At the same time, these countries are more likely to have ongoing BCG vaccination programs, given the higher level of tuberculosis burden [7].

Escobar et al. considered the number of deaths instead of the number of cases, which could, to some extent, compensate for inequality in testing capacity between the analyzed countries. Recent research based on the “excess death” approach suggests that COVID-19 death tolls are likely to be underestimated, by around 30% of COVID-19 deaths across the United States from 1 March to 30 May 2020 [8]. The reasons may include delays in COVID-19 death reporting, as well as the misattribution of COVID-19 deaths to other illnesses either resembling COVID (pneumonia) or reflecting its complications (myocarditis, coagulopathy) [9].

These findings reveal that estimating the COVID-19 death toll considering excess all-cause mortality may be more reliable than being based only on reported deaths, especially in countries with an insufficient testing capacity [8].

Although data indicate a correlation between BCG vaccination policy and COVID-19 mortality, the confirmed deaths rates vary remarkably among the countries with the same BCG policy. The variability between countries with high BCG coverage might be explained, at least in part, by different BCG strains used for inducing trained immunity [10]. The currently used BCG vaccine was initially developed at the Pasteur Institute, Paris, in 1921, through the attenuation of a *Mycobacterium bovis* strain isolated from cattle with tubercular mastitis [10]. The original strain was distributed to separate laboratories worldwide, and a number of subsequent strains were then produced by serial passage. These strains differ genetically and phenotypically and have different viable bacterial counts and reactogenicity profiles [4]. Previous studies [4,10] have shown that countries adopting BCG-Japan or a mixture of different BCG vaccine strains reported a lower number of confirmed and fatal COVID-19 cases. By contrast, countries that use BCG-Denmark and BCG-Brazil performed worse in terms of COVID morbidity and mortality compared with other countries with current BCG vaccination [4,10].

However, even if BCG vaccination does contribute to reducing COVID-19 mortality, it is definitely not the only factor, as can be illustrated by Australia and Finland’s cases, which report low COVID-19 deaths despite refusing mandatory BCG vaccination [4].

## 2. The Specificity of SARS-CoV-2—Molecular Characteristics

The causative agent of SARS-CoV-2 is a virus belonging to the *Orthocoronavirinae* subfamily, a member of the *Coronaviridae* family. The *Orthocoronavirinae* subfamily includes the following genera: *Alphacoronavirus, Betacoronavirus, Gammacoronavirus,* and *Deltacoronavirus* [11]. The genera *Alphacoronavirus* and *Betacoronavirus* are further subdivided into subgroups 1a–1b and 2a–2d, respectively [12]. The genetic material of SARS-CoV-2 is larger than other known RNA viruses and is a single-stranded positive-sense RNA (+ssRNA) of 29.9 kb [13]. Viruses belonging to this family cause infections in birds [14] and mammals of various species [15]. First described in 1965, coronavirus affects the human respiratory tract, mainly causing mild respiratory symptoms, such as HCoV-NL63, HCoV-229E, HCoV-OC43, and HCoV-HKU1 [15,16,17]. However, coronavirus infections in humans can also cause severe respiratory symptoms, as could previously be observed in 2002 in China (SARS-CoV) [18] and in 2012 in Saudi Arabia (MERS-CoV) [19], causing the death of approximately 800 people in both cases. Among the seven human coronaviruses (HCoVs) described so far, the pandemic caused by SARS-CoV-2 is the largest yet.

The similarity of the SARS-CoV-2 nucleotide sequence to previously described coronaviruses causing acute respiratory symptoms in humans is not high, at 79% and 50% to SARS-CoV and MERS-CoV, respectively. The newly described SARS-CoV-2 shows the highest genetic similarity, of approximately 90%, to bat-SL-CoVZC45 and bat-SL-CoVZXC21 animal coronaviruses [16] and 96% to BatCov RaTG13 [20]. The homology to SARS-CoV of about 80% resulted in SARS-CoV-2 also having ACE2 as the receptor to which the virus binds during infection [16,21,22], although it is also characterized by greater infectivity and transmissibility [23,24].

**Figure 1:**
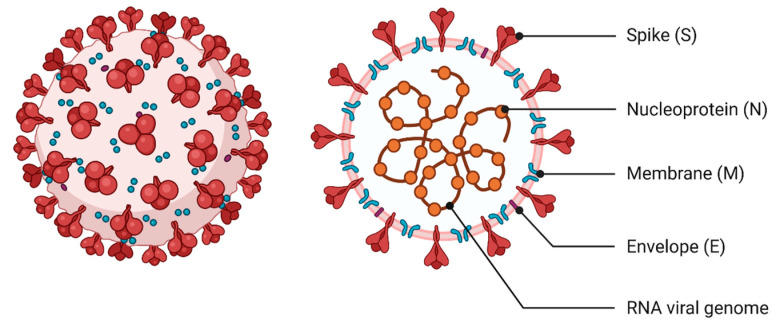
Human SARS-CoV-2 structure. SARS-CoV-2 is composed of 4 structural proteins: membrane protein (M), spike protein (S), envelope protein (E) and nucleoprotein (N), which is responsible for the formation of the capsid. The genetic material of SARS-CoV-2 is a single-stranded positive-sense RNA. Created with BioRender.com (accessed on 8 May 2021).

The viral genome consists of 10 open reading frames (ORFs), of which ORFa/ab encodes the two pp1a and pp1b polyproteins, while ORFs 2–10 are responsible for encoding structural and accessory proteins [23]. SARS-CoV-2 is composed of four structural proteins: membrane protein (M), spike protein (S), envelope protein (E) and nucleoprotein (N), which is responsible for the formation of the capsid (Figure 1). The structural genes, except the S gene, show 90% amino acid sequence similarity to SARS–CoV [16]. In addition, there are 16 non-structural proteins (nsp1–16) that are relevant to pathogenesis and arise from the pp1a and pp1b polyproteins [17,25]. Analogous to other beta coronaviruses, accessory proteins 3a, 6, 7a, 7b, 8 and 10 have also been demonstrated [26].

## 3. The Specificity of SARS-CoV-2 Infection and Host Reaction—Clinical Approach

The virus is mainly transmitted by droplet contact between symptomatic and asymptomatic infected and uninfected persons. However, the possibility of SARS-CoV-2 transmission through indirect contact, as a result of contamination with infectious agents, has also been demonstrated [25]. A very important route of infection can also be through aerosol, making it important to employ protective measures, such as facemasks to avoid infection [27,28]. Furthermore, fecal–oral transmission, especially in children, cannot be excluded [29], and the presence of viral particles in the urine of COVID-19 patients has also been described [30]. Previously, it has been assumed that infections by the vertical route do not occur in pregnancy [31,32], although a number of studies have recently shown that this low possibility of such transmission exists [33,34]. Interestingly, the possibility of transmission of infection from human to animal, including tiger, dog and cat, has also been demonstrated [35,36,37,38]. To date, the possibility of infection in the reverse direction, that is, from animal to human, has not been described [37]. Although, in the context of animals as intermediate hosts for HCoVs, bats and pangolins are probably the greatest danger [20,39,40].

**Figure 2:**
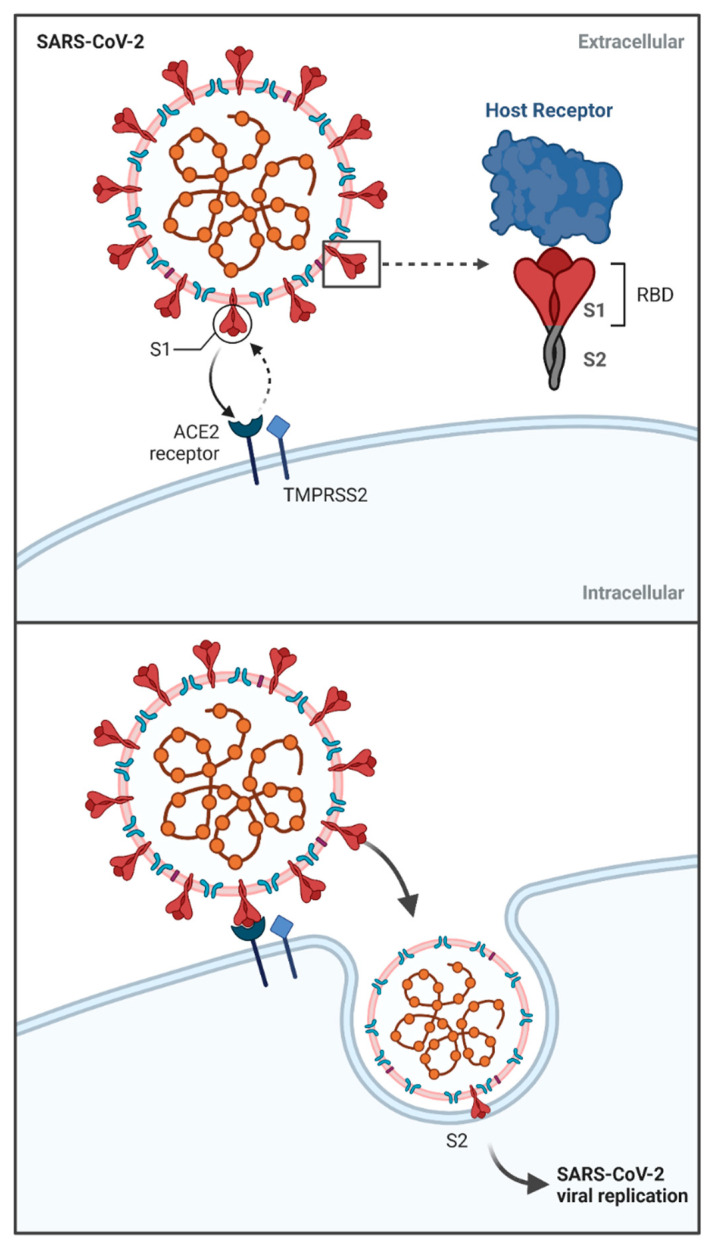
Mechanism of SARS-CoV-2 viral entry. Viral S glycoprotein contains two subunits: S1 and S2, with S1 constituting receptor binding domain (RBD). S1 subunit associates with host cell’s receptor ACE2 with the participation of TMPRSS2, which leads to conformational changes in S1 subunit and endosomal reaction. Subsequently, S2 subunit activates host cell fusion with viral particles, leading to viral replication. Abbreviations: ACE2—angiotensin-converting enzyme 2; RBD—receptor binding domain; TMPRSS2—transmembrane protease serine 2. Created with BioRender.com (accessed on 8 May 2021).

The fusion of a specific receptor with a virus particle allows the viral life cycle to take place, which leads to the production of new infective forms. The binding site of the SARS-CoV-2 molecule in the host is angiotensin-converting enzyme 2 (ACE2) [41] (Figure 2). The same binding site is shown by previously described SARS-CoV [42], while the receptor for MERS-CoV was identified as dipeptidyl peptidase-4 (DPP4, also known as CD26) [43]. Hence, the number and distribution of the above described receptors in the host influence the pathogenicity and intensity of symptoms after coronavirus infection [44].

ACE2 receptors for SARS-CoV-2 are mainly distributed in the airway epithelium, hence the characteristics of the disease symptoms are related to this tract [45]. The presence of these receptors also in the small intestinal epithelium, kidney, and brain could potentially be the site of SARS-CoV-2 binding and induction of disease symptoms [46,47]. Additionally, they are found in many tissues of the body, including the vascular endothelium and cells associated with the immune system (macrophages and monocytes) [46,47,48]. ACE2 has been described in many mammalian species, implicating the ability of CoV2 to break the species barrier during SARS infection [49]. Interestingly, interferons I and II produced in viral infections have been shown to affect the increased expression of ACE2 receptors in human airways [50].

In the case of MERS-CoV, in vitro studies have shown the possibility of infecting dendritic cells and even T lymphocytes, thus causing limited antiviral activity of the host organism. Such activity of MERS-CoV virus is connected with its affinity to the CD26 receptor [51,52,53].

Additionally, important elements involved in the activation of viral infection are proteases: transmembrane protease serine 2 (TMPRSS2), cathepsin B (CatB) and cathepsin L (CatL) [54,55,56]. The occurrence of these proteases, especially TMPRSS2, is closely correlated with the course of SARS-CoV-2 infection. The role of these enzymes has been confirmed in studies showing that their inhibition induced by camostat mesylate and nafamostat mesylate results in a reduction in coronavirus pathogenicity [57,58].

Proteolytic enzymes are closely involved in the cleavage of coronavirus S glycoprotein [55,56]. Structural protein S is the most essential element of the viral particle, involved in viral entry into host cells, containing the receptor binding domain (RBD) [17,59]. The Leu455, Phe486, Gln493, Ser494, Asn501, and Tyr505 amino acids within the RBD have been shown to be most involved in ACE2 binding [60]. This S protein is composed of 1273 amino acids, S1 and S2 subunits distinguished within. The S1 subunit is responsible for the association of the viral particle with the host cell via the ACE2 receptor, after which the conformational changes in the protein initiate the endosomal reaction mediated by clathrin-dependent and -independent endocytosis [21,61] (Figure 2). Numerous conformational changes of the S protein were also confirmed in relation to the S2 subunit, whose role in SARS-CoV-2 infection is to activate host cell fusion with viral particles [17]. The relationships between protein S and the host cell described above indicate the high complexity of this process, the understanding of which is necessary to establish therapeutic options against SARS-CoV-2 [62]. It is noteworthy that the S protein responsible for binding to ACE2 shows great variability in structure, in particular the RBD fragment. This is associated with the occurrence of mutations within it, which may cause variability in the antigenicity of SARS-CoV-2. In addition, the described variability may also influence the widening of the infectious spectrum by breaking the species barrier during infection [63,64,65]. S-glycoprotein is mainly responsible for the activation of immune processes related to specific and innate immunity [66].

Another structural protein important for SARS-CoV-2 infection is the envelope membrane (E) protein. This protein is responsible for the formation of viral particles by participating in ion channels, but it also partly modulates pathogenesis. Therefore, this small protein (75 aa) may be an important target in the context of anti-SARS-CoV-2 therapy [67].

Other structural proteins, membrane protein (M) and nucleocapsid protein (N), are responsible for the transcription, translation and packaging of viral RNA and the formation of viral particles. The M protein is composed of 222 and the N protein of 419 amino acids. In addition, these M and N proteins share high structural similarity with previously described coronaviruses. Therefore, it is feasible to exploit this feature for therapeutic purposes, in which antibodies to the N protein produced during SARS-CoV infection could be effective against SARS-CoV-2 [68,69].

In addition to the four structural proteins described, there are also 16 non-structural proteins (NSPs) within the virus. They have numerous functions associated with viral infection, including its replication [16,22]. Additionally, NSPs are responsible for the early antiviral response, also influencing the pathogenicity of the virus through immunomodulation and regulation of gene transcription [70,71]. More specifically, Nsp1 is involved in viral replication by participating in translation and mRNA degradation. In turn, non-structural protein 2 is involved in the cellular signaling of host cells, while Nsp3 and Nsp5 are responsible for proteolytic processes occurring within the produced polyproteins. Furthermore, Nsp4 affects ER membranes and thus influences viral replication, and non-structural protein 6 initiates the formation of autophagosomes from the endoplasmic reticulum. Nsp7 is an RNA-dependent RNA polymerase having a major effect on virus replication, similar to non-structural proteins 8, 9, 12, 13. Nsp10 is involved in the activation of virus transcription and, like Nsp16, has methyltransferase-like activity. Nsp14 and Nsp15 are nucleases that act within the genetic material of the virus. The function of non-structural protein 11 has not yet been understood. All of the above described activities of the structural proteins are complementary and enable the following steps necessary for virus replication to occur [17].

## 4. Genetic Variability of SARS-CoV-2

An important feature of coronaviruses is their high variability in genetic material, which can result in the infection of different animal species [72], as has already been demonstrated with HCoV [12,15]. Additionally, this is confirmed by studies on HCoV-OC43s, which was described in 1967 and is still circulating in the environment [73,74,75]. On the basis of these studies [73], four genetic subgroups have been described, which show nucleotide variation in nt 23,449 to 26,332, which is important in creating an effective vaccine against SARS-CoV-2.

**Figure 3:**
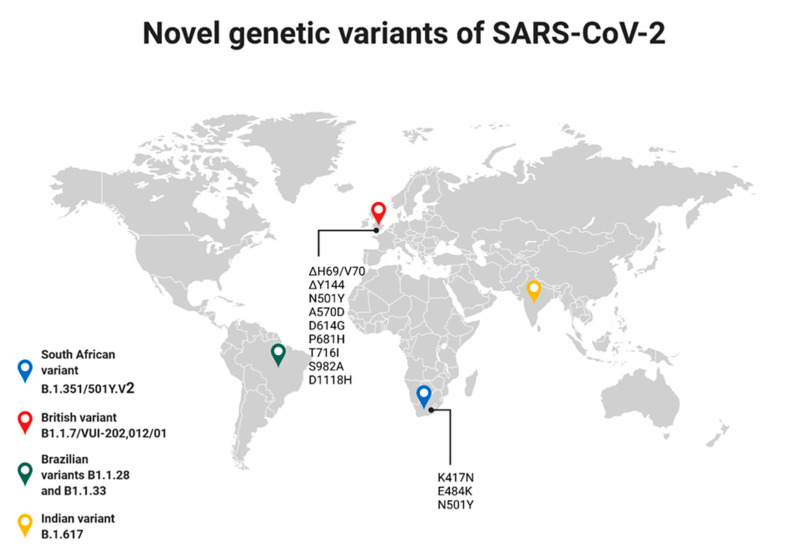
Novel genetic variants of SARS-CoV-2 have been described and their origin is marked on the map. Additionally, mutations responsible for the occurrence of British and South African variants are marked. Created with BioRender.com (accessed on 8 May 2021).

The greatest genetic variation in SARS-CoV-2 has been demonstrated within the structural protein S. The recently described UK variant B1.1.7/VUI-202,012/01 shows multiple mutations in its spike (S) protein: ΔH69/V70, ΔY144, N501Y, A570D, D614G, P681H, T716I, S982A, and D1118H (H, His; V, Val; Y, Tyr; N, Asn; A, Ala; D, Asp; G, Gly; P, Pro; T, Thr; I, Ile; S, Ser). It is noteworthy that the N501Y mutation was associated with an expansion of the infectious spectrum of the virus to the mouse [76]. This variation is also associated with a substitution at the 614 amino acid of the S protein (Asp614-to-Gly), showing high prevalence worldwide, “displacing” the original SARS-CoV-2. As a result of this mutation, a variant of the D614G virus has been described that may be associated with altered virulence, pathogenicity and transmission of this pathogen in the environment [77,78]. In vitro, this variant showed more intense infection and replication compared to the original virus. As a result, the variant shows faster proliferation in the environment. The morphology of the compared viruses and their neutralization in vitro were similar, suggesting that vaccines against SARS-CoV-2 may be effective against the described British variant [79,80,81]. SARS-CoV-2 variants have been shown to undergo differential glycosylation, which is important with respect to their infectivity and antibody reactivity. In addition, many other virus variants have been described, including A475V, L452R, V483A, F490L and N234Q, which are less infectious but more resistant to antibodies, which may be important for vaccine efficacy and should be carefully studied [82]. A recent case of SARS-CoV-2 reinfection in a 25-year-old male has been described, confirmed by testing for genetic material from the virus as being a different variant from that causing the first infection [76]. This situation may raise many questions about the effectiveness of vaccination against SARS-CoV-2, as its genetic variability may randomly affect its characteristics related to the development of the epidemic. A second described variant of SARS-CoV-2 is B.1.351/501Y.V2, originating from South Africa [83]. It is characterized by three mutations within the S protein: K417N, E484K, as well as N501Y, analogous to the British variant mutation [84,85]. Since it was first described (November 2020) it has spread to many countries, including Brazil, France, Australia, Germany, Switzerland, Japan, Sweden, South Korea, Finland, Ireland, the Netherlands, and the UK [83,85]. This demonstrates its high transmissibility, as evidenced by the fact that within a few weeks it had become the main causative agent of SARS-CoV-2 in people living in southern Africa [85]. The next significant SARS-CoV-2 variants described are B1.1.28 and B1.1.33, originating from Brazil [86]. The presence of these variants in the environment has resulted in a much faster spread of the virus within Brazil, making it the third country in the world for SARS-CoV-2 infections [87]. Furthermore, the variant spreading very rapidly in India has been described as the latest SARS-CoV-2 virus variant, B.1.617, causing about 400,000 cases per day by the end of April 2021 [87], and it is now the predominant causative agent of COVID-19 [88]. Due to mutations in this variant, it has been suspected that vaccines may be ineffective against it, but this has not been confirmed [89]. Analyzing the rate of virus spread in the world and the number of cases of infection in individual countries, it is possible to notice a correlation that a significant increase in the number of described cases is associated with the appearance of SARS-CoV-2 variants in a given region of the world [87] (Figure 3).

The described mutations mainly affect the rate of spread of the virus, without much influence on its pathogenicity and the effectiveness of vaccination against it [79,80,81]. In addition, the mutations are within the ORF3a protein, which may be involved in the pathogenesis of the virus [90]. Summarizing, the described mutations should be taken into account from the point of view of understanding the biology of the virus and the related possibilities in the production of effective vaccines.

## 5. COVID-19—The Current Knowledge of the Pandemic and the Disease

The number of people affected by the current pandemic continues to increase intensively (especially in India), with about 150 million infections worldwide and over 3 million deaths [87]. Compared to earlier human coronaviruses, this pandemic has assumed skyrocketing proportions. The daily peak of SARS-CoV-2 cases worldwide was recorded in early January 2021 (it was almost 850 thousand cases), while at the end of April 2021 (29 April) it amounted to more than 900 thousand cases [87]. Large variation in the number of cases in different parts of the world is described, related to the intensity of vaccination against SARS-CoV-2 carried out by different countries and resulting from the fact that less wealthy countries, where the rate of vaccination is lower, face much higher dynamics of viral spread. In addition, the higher rate of infections of this disease in the environment is influenced by the described variants of the virus, which are characterized by greater pathogenicity and ability to spread. Studies on the efficacy of monoclonal antibodies produced after the application of commercially available vaccines seem to be optimistic, showing that they should be effective against the newly emerged variants [79,80,81].

However, because of the characteristic variability described within the RNA of viruses, including coronaviruses, it is important to keep in mind possible changes in their pathogenicity. In view of this aspect, it is necessary to carry out precise studies on the sequence of the genetic material of SARS-CoV-2 and the associated possibility of new viral feature acquisition. Therefore, it seems necessary, in addition to the use of immunoprophylaxis, to find new therapeutic agents that can be used in the treatment of patients with COVID-19. Particularly noteworthy are the reports on the use of somatic mesenchymal cells in therapy against COVID-19 [91,92,93,94]. These studies show that the use of MSCs derived from perinatally obtained tissues in patients with severe symptoms of COVID-19 was safe, resulting in improved results of clinical examinations. However, while the efficacy of the use of MSCs in imaging studies (CT) has been confirmed, more thorough research in this area is required [91,92,93,94].

When describing COVID-19, three different types of disease intensity are often denoted: moderate, severe and critical [25]. The intensity of clinical symptoms depends on many factors, which are mainly related to the age and health status of patients [31]. Research to date indicates that people of any age can be infected with SARS-CoV-2, although the average COVID-19 patient age is 50 years [25], with particular severity in those affected by comorbidities [31]. Severe infection is more common in people over the age of 60, with 8% mortality rate in those aged 70 and 79, and 14.8% for those over 80 years of age [95]. In addition, the comorbidities that affect higher mortality due to SARS-CoV-2 include conditions such as hypertension, cancer, diabetes, vascular and circulatory diseases and chronic respiratory diseases [31]. Clinical symptoms of COVID-19 are mainly associated with fever, dry cough, fatigue, shortness of breath, muscle ache, confusion, headache, sore throat, rhinorrhea, olfactory and taste disorders and additionally with chest pain, diarrhea, nausea, vomiting, chills, sputum production, hemoptysis, dyspnea, bilateral pneumonia, or anorexia. SARS-CoV-2-induced symptoms are also characterized by changes in laboratory parameters causing leukopenia, lymphopenia, and higher levels of plasma cytokines (IL2, IL7, IL10, GSCF, IP10, MCP1, MIP1A, and TNFα) [25].

## 6. The Characteristic of BCG Strains

Vaccination against tuberculosis (TB) has been used for more than a century. It is the most widely used vaccine in the world with over 4 billion vaccinations to date [96]. The live TB vaccine currently in use is the Bacillus Calmette–Guérin (BCG) [97]. There are six strains within this vaccine, which include the Pasteur 1173 P2, the Danish 1331, the Glaxo 1077 (derived from the Danish strain), the Tokyo 172-1, the Russian BCG-I and the Moreau RDJ, all of which exhibit varying immunogenicity [97]. Vaccines containing the Glaxo 1077, Tokyo 172-1, or Moreau RDJ strains have been shown to cause fewer side effects than the others. In addition, the amount of live antigens contained in a single dose varies from strain to strain and ranges from 50,000 to 3,000,000 [97]. Due to the large-scale use of TB vaccines (100 million per year), there is a need for new vaccines, of which there are about 20 in production and at various stages of development [96,98,99]. These new TB vaccines fall into three groups: live-attenuated, inactivated whole cell and subunit vaccines. The live-attenuated vaccines VPM1002 and MTBVAC are the most advanced, with positive results from preliminary clinical trials already published [100,101].

**Figure 4:**
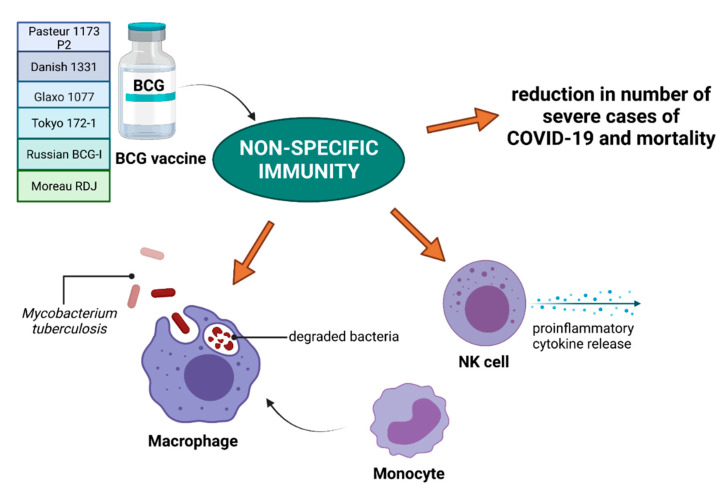
BCG vaccine against tuberculosis includes six strains: Pasteur 1173 P2, Danish 1331, Glaxo 1077, Tokyo 172-1, Russian BCG-I and Moreau RDJ. Apart from preventing tuberculosis, BCG vaccine exhibits immunomodulatory effects, resulting in activation of non-specific immunity, macrophage activation and proinflammatory cytokine release by NK cells. Such response after vaccination was shown to be associated with reduction in severe cases of COVID-19 and COVID-19-related mortality. Abbreviations: BCG—Bacillus Calmette–Guérin; NK—natural killer. Created with BioRender.com (accessed on 8 May 2021).

The BCG vaccine is effective against tuberculosis, but many studies have shown its strong immunomodulatory effect, important in terms of immunity to other diseases [102,103,104,105]. Its action leads to a decrease in mortality, especially in children, caused by various pathogens causing, e.g., respiratory infections [106]. The effect of the BCG vaccine is the activation of non-specific immune memory induced after vaccination, known as “trained immunity” [107] (Figure 4).

This type of immunity is mainly dependent on cells associated with the organism’s non-specific immunity, including macrophages, monocytes and NK cells (natural killers) [104,106,107]. This stimulation of the immune system may also affect the course of SARS-CoV-2 infection, which has recently been investigated by a number of researchers [1,2,4,106,107,108,109,110,111,112]. The results of these studies show that the stimulation of the immune system after BCG vaccine administration results in a reduction in the number of severe cases, as well as reduces mortality due to the stimulation of non-specific immunity [1,108,110,112]. Hence, countries with universal TB vaccination have been shown to have lower rates of COVID-19 infection and mortality associated with this disease [10]. Interestingly, the type of strain used in the BCG vaccine has a described impact on the immunity to SARS-CoV-2. However, BCG vaccines containing BCG-Russia, BCG-Denmark, and BCG-Brazil strains have been shown to induce significantly weaker immunity to COVID-19 compared to BCG-Japan, BCG-Pasteur and BCG-Mix strains [10]. This relationship was confirmed by both the number of infections and mortality in countries using different types of BCG vaccines. However, studies indicating that universal BCG vaccination policy in India results in significant reductions in COVID-19 cases are currently unconfirmed [87,108]. Furthermore, this theory is supported by the results of epidemiological studies showing that the BCG vaccine does not induce protection against COVID-19 [109,111]. Hence, it can be hypothesized that in the initial stage of the pandemic, countries with universal BCG vaccination policies (India, Poland) showed a low rate of virus spread and low mortality among the infected [10,87]. Such studies were particularly important until the introduction of COVID-19 vaccines, which provide protection after the development of specific immunity. However, with the development of the pandemic and the emergence of new variants of the virus, infection rates still increased significantly [87,88]. It is possible that emerging mutations within SARS-CoV-2 affect the pathogenicity of the virus and its ability to spread in the environment despite the “trained immunity” created by BCG vaccination.

## 7. The BCG Vaccination—Current Strategies and WHO Recommendations

Over the years, TB vaccination policies have changed in many countries. However, in the era of large migration of people, especially from countries with undefined vaccination policies, to other countries, a risk analysis should be performed. Some countries have stopped vaccination because of the low number of TB cases, which is a change in public health policy, or the presence of other infectious diseases (for example, HIV). Other countries have maintained mandatory vaccination for certain high-risk groups of people. Such variability in vaccination policy also concerned the number of doses, the route of administration (mainly intradermal) and the age at which people were immunized [113], and especially that the efficacy of TB vaccines in adults shows a high variability [114]. The genome variation of vaccines over 100 years also affects immunogenicity and the response to intradermal tuberculin testing [115]. Current TB vaccination policy in the world distinguishes countries that: used to vaccinate all people and do not currently do so, vaccinate only certain groups of people at significant risk for tuberculosis, and those that vaccinate all people, currently the most numerous group [116]. Using the available data from 180 countries, it is registered that 157 countries have mandatory vaccinations; the remaining 23 countries have discontinued mandatory vaccinations or only vaccinate certain risk groups. Regarding the number of doses administered, in the vast majority of countries a single vaccination is performed. Some countries (16 countries, previously 33 countries) also perform a second vaccination to achieve a booster effect. Countries such as Kazakhstan, Belarus, Uzbekistan, and Turkmenistan perform an additional 3rd vaccination between the ages of 12 and 15 years. As far as the age of immunized persons is concerned, the first TB vaccination is usually administered on the 1st day of life, while countries that administer the second vaccination perform it at the age of about 7 years. Currently, eight countries perform TST (tuberculin skin test) post first BCG vaccination and depend on it for the necessity of performing the second vaccination. The amount of TB vaccination administered by individual countries over the years has been intensively changing, leading mainly to a single vaccination policy. In addition, changes in vaccination policies relate to the BCG strains used, where a large number of countries use multiple BCG strains, which may affect the effectiveness of immunization [113].

Due to the high number of deaths caused by tuberculosis worldwide, the WHO recommends subjecting the general population to BCG vaccination. This recommendation is particularly applicable to developing countries with high TB seroprevalence, but also countries with high rates of leprosy and Buruli ulcer [117]. This vaccination should be given to healthy children on day 1 of life or at the earliest opportunity [118]. Additionally, combining the TB vaccine with the hepatitis B vaccine is highly recommended, and use with other routine vaccinations in children is not a problem [118]. Countries conducting TB vaccination selectively should carefully analyze the epidemiological status of newborns. The decision for a country to switch from a mandatory to a fringe risk vaccination policy should be made after conducting epidemiological studies that also include leprosy. For older children, TB vaccination should be administered to TST- or IGRA-negative children who move from high TB/leprosy-prevalence areas, from low to high TB/leprosy-prevalence areas, or to older children at risk of TB/leprosy (risk fringe) [117]. The WHO does not recommend performing revaccination, even if TST- or IGRA-negative, due to lack of evidence of increased protection against TB/leprosy [119]. Additionally, the absence of a vaccination scar does not indicate a lack of acquired immunity [117]. TB vaccination should not be performed in pregnant women, HIV/AIDS patients [118], low birth weight preterm infants, or persons on immunosuppressive therapy. In addition, persons traveling to countries with a high incidence of the disease should be vaccinated. BCG vaccines should be given by intradermal injection at a dose of 0.05 mL for children up to 1 year of age and 0.1 mL over 1 year of age [117]. It is worth adding that BCG vaccination causes the previously mentioned immunomodulation that also protects against other groups of pathogens [120].

## 8. Conclusions

The high genetic variability described within SARS-CoV-2, manifested by the description of new viral variants, should be carefully analyzed. Based on the data presented here, it can be seen that in the early stages of the pandemic, BCG vaccination used on a large scale in selected countries resulted in lower mortality in those countries due to COVID-19. This described immunity is due to the immunomodulatory effect of the vaccine used against tuberculosis and also depends on the BCG strain. However, over time, the description of new SARS-CoV-2 variants has increased the mortality rate in these countries. Finally, it was shown that there was no effect of TB vaccination on mortality caused by COVID-19, regardless of the strain used in the BCG vaccine. This may be due to the adaptation of the virus to adverse host conditions. Presumably, SARS-CoV-2 has adapted to the immunity resulting from vaccination with the less immunomodulatory strains BCG-Denmark and BCG-Brazil, and subsequently also with the BCG-Japan strain, causing the absence of any effect of BCG vaccination on SARS-CoV-2. The relationship between COVID-19 and BCG requires further study, but confirms the ability of SARS-CoV-2 to adapt to adverse conditions. In addition, the described features of RNA viruses associated with their intensive genetic variability may result in the demonstration of features associated with their pathogenicity, making it important to continue research in this direction.
